# Impact of medical and psychiatric multi-morbidity on mortality in diabetes: emerging evidence

**DOI:** 10.1186/1472-6823-14-68

**Published:** 2014-08-20

**Authors:** Cheryl P Lynch, Mulugeta Gebregziabher, Yumin Zhao, Kelly J Hunt, Leonard E Egede

**Affiliations:** 1 Health Equity and Rural Outreach Innovation Center, Charleston VA HSR & D COIN, Charleston, SC, USA; 2 Department of General Internal Medicine & Geriatrics, Center for Health Disparities Research, Medical University of South Carolina, 135 Rutledge Avenue, MSC 593, Charleston, SC, USA; 3 Department of Public Health Sciences, Medical University of South Carolina, Charleston, SC, USA

**Keywords:** Diabetes, Comorbidity, Mortality, Elderly, Veterans

## Abstract

**Background:**

Multi-morbidity, or the presence of multiple chronic diseases, is a major problem in clinical care and is associated with worse outcomes. Additionally, the presence of mental health conditions, such as depression, anxiety, etc., has further negative impact on clinical outcomes. However, most health systems are generally configured for management of individual diseases instead of multi-morbidity. The study examined the prevalence and differential impact of medical and psychiatric multi-morbidity on risk of death in adults with diabetes.

**Methods:**

A national cohort of 625,903 veterans with type 2 diabetes was created by linking multiple patient and administrative files from 2002 through 2006. The main outcome was time to death. Primary independent variables were numbers of medical and psychiatric comorbidities over the study period. Covariates included age, gender, race/ethnicity, marital status, area of residence, service connection, and geographic region. Cox regression was used to model the association between time to death and multi-morbidity adjusting for relevant covariates.

**Results:**

Hypertension (78%) and depression (13%) were the most prevalent medical and psychiatric comorbidities, respectively; 23% had 3+ medical comorbidities, 3% had 2+ psychiatric comorbidities and 22% died. Among medical comorbidities, mortality risk was highest in those with congestive heart failure (hazard ratio, HR = 1.92; 95% CI 1.89-1.95), Lung disease (HR = 1.42; 95% CI 1.40-1.44) and cerebrovascular disease (HR = 1.39; 95% CI 1.37-1.40). Among psychiatric comorbidities, mortality risk was highest in those with substance abuse (HR = 1.50; 95% CI 1.46-1.54), psychoses (HR = 1.16; 95% CI 1.14-1.19) and depression (HR = 1.05; 95% CI 1.03-1.07). There was an interaction between medical and psychiatric comorbidity (p = 0.003) so stratified analyses were performed. HRs for effect of 3+ medical comorbidity (2.63, 2.66, 2.15) remained high across levels of psychiatric comorbidities (0, 1, 2+), respectively. HRs for effect of 2+ psychiatric comorbidity (1.69, 1.63, 1.42, 1.38) declined across levels of medical comorbidity (0, 1, 2, 3+), respectively.

**Conclusions:**

Medical and psychiatric multi-morbidity are significant predictors of mortality among older adults (veterans) with type 2 diabetes with a graded response as multimorbidity increases.

## Background

Multi-morbidity, or the presence of multiple chronic diseases, is a major problem in clinical care and is associated with worse outcomes [1-3]. People with multi-morbid conditions also have a higher medication burden [4] and, not unexpectedly, have worse medication adherence [5]. The prevalence of multi-morbidity is high (>50%) in middle-aged and older populations [6]. However, most health systems are generally configured for management of individual diseases instead of multi-morbidity. A recent review article suggests the number of studies testing interventions for patients with multi-morbid disease has increased, but the findings showed that organizational interventions with a broader focus and patient-specific interventions not linked to care delivery had little impact on health outcomes [7].

The presence of mental health conditions (e.g., depression, anxiety, etc.) in people with multi-morbidity has adverse impact on clinical outcomes [8,9]. A study of the impact of psychiatric conditions (depression/anxiety, substance abuse, psychotic or bipolar disorder) on mortality among individuals with diabetes indicated that alcohol and drug abuse/dependence was associated with a 22% higher mortality [10]. Chronic conditions like diabetes tend to occur in clusters (with other cardiovascular conditions like hypertension, heart disease and stroke) [11]. However, very little is known about the separate and combined impact of medical and psychiatric conditions on mortality and which has the strongest impact on risk of death in individuals with diabetes. Few studies have carefully assessed the impact of both medical and psychiatric multi-morbidity on mortality in middle-aged and older adults with diabetes.

Older aged and elderly individuals have greater disease burden and are more likely to have multimorbidity and experience the adverse outcomes associated with multimorbidity. Because of the detailed clinical data available in the Veterans Health Administration (VHA) databases, the older age, and the ability to track patients over long periods of time, veterans are an ideal population to tease apart the impact of medical and psychiatric multi-morbidity on mortality. However, the focus of this study is to examine the impact of total burden of disease rather than specific comorbidities, so we used a national sample of veterans with type 2 diabetes followed over 5 years to examine the prevalence and differential impact of medical and psychiatric multi-morbidity on risk of death.

## Methods

### Study population

A national cohort of veterans with type 2 diabetes was created by linking multiple patient and administrative files from the VHA National Patient Care and Pharmacy Benefits Management (PBM) databases. We used a previously validated algorithm [12,13] for identifying veterans with diabetes. Veterans were included in the cohort if they had: 1) type 2 diabetes defined by two or more International Classification of Diseases, Ninth Revision (ICD-9) codes for diabetes (250, 357.2, 362.0, and 366.41) in the previous 24 months (2000 and 2001) and during 2002 from inpatient stays and/or outpatient visits on separate days (excluding codes from lab tests and other non-clinician visits); or 2) prescriptions for insulin or oral hypoglycemic agents (VA classes HS501 or HS502, respectively; to capture those without a diabetes ICD-9 code) in 2002. PBM data were available during the entire period of analysis. When the data were merged based on the criteria above, the total sample included 832,000 veterans. We excluded those not taking prescription medications for diabetes (n = 201,255) and added those who had one ICD-9 code for diabetes and prescriptions filled in 2002 (n = 60,493); and 3,660 were excluded due to death prior to 2002, missing age or no service connection. The subset with complete data resulted in a final cohort of 625,903 veterans. The Department of Veterans Affairs maintains data through the VA Information Resource Center (VIReC). Data was requested from and approved for use by VIReC following data use agreement requirements. The study was approved by the Medical University of South Carolina Institutional Review Board (IRB) and the Ralph H. Johnson Veterans Affairs Medical Center Research and Development committee.

### Outcome measure

The main outcome measure was time to death. Veterans were followed from time of entry into the study until death, loss to follow-up, or through December 2006. A subject was considered censored if alive by December 2006.

### Primary covariates

The primary covariates were medical comorbidity and psychiatric comorbidity both defined as the count of diseases for each subject throughout the study period. All comorbidities were dichotomized as present or absent where presence was determined by ICD-9 codes at entry into the cohort based on a previously validated algorithm in veterans [13]. Medical comorbidity variables included anemia, cancer, cardiovascular disease (CVD), cerebrovascular disease, congestive heart failure (CHF), fluid and electrolyte disorders, hypertension, hypothyroidism, liver disease, lung conditions (chronic pulmonary disease, pulmonary circulation disease), obesity, peripheral vascular disease, and other (acquired immunodeficiency syndrome–AIDS, rheumatoid arthritis, renal failure, peptic ulcer disease and bleeding, weight loss). Psychiatric comorbidities included psychoses, substance abuse (alcohol abuse, drug abuse) and depression [13].

### Demographic variables

We controlled for seven demographic variables. Age was treated as continuous and centered at a mean of 66 years. Race/ethnicity included four categories with non-Hispanic white (NHW) serving as the reference group. Race/ethnicity was retrieved from the 2002 outpatient and inpatient [Medical SAS] data sets. When missing or unknown, the variable was supplemented using the inpatient race1-race6 fields from the 2003 [Medical SAS] data sets, the outpatient race1-race7 fields from the 2004 [Medical SAS] data sets, and the VA Vital Status Centers for Medicare and Medicaid Services (CMS) field for race. Gender, marital status, and location of residence (urban versus rural or highly rural) were dichotomous. Highly rural was categorized as rural according to the VA definition of rurality [14]. Percentage service-connectedness, representing the degree of disability due to illness or injury that was aggravated by or incurred in military service, was treated as dichotomous (1= > 50%, 0 = <50%). Region, which accounts for the five geographic regions of the country, was treated as a categorical variable: Northeast [VISNs 1, 2, 3], Mid-Atlantic [VISNs 4, 5, 6, 9, 10], South [VISNs 7, 8, 16, 17], Midwest [VISNs 11, 12, 15, 19, 23], and West [VISNs 18, 20, 21, 22] [15].

### Statistical analysis

In preliminary analyses, crude associations were examined between mortality and all measured covariates using chi-square tests for categorical variables and t-tests for continuous variables. Cox regression methods were used to model the association between time to death and medical and psychiatric comorbidity after adjusting for known covariates. Time to death was defined as the number of months from time of entry into the cohort to time of death or censoring (i.e., day last seen or May 2006). For the Cox model, appropriateness of the assumption of proportionality was determined by testing the coefficients of the interactions of time with the respective covariate in multivariate analyses. Initially, Cox models for each of the medical and psychiatric comorbidities were fitted adjusting for all covariates (race, socio-demographics). Then an interaction between medical and psychiatric comorbidity was tested to check whether the association between mortality and medical comorbidity was modified by the presence of psychiatric comorbidity. HR estimates of medical comorbidity for each level of psychiatric comorbidity and estimates for levels of psychiatric comorbidity for levels of medical comorbidity are reported since there was significant interaction (p = 0.003). The Kaplan-Meier method was used to plot the survival functions for both medical and psychiatric comorbidities separately. Residual analysis was used to assess goodness-of-fit of each of the models. All data analyses were conducted using SAS 9.3 [16].

## Results and discussion

The study population consisted of 625,903 veterans with diabetes who were followed until death, loss to follow-up, or through December 2006. The mean age was 65.4 years (sd 11.1) with a range of 18–100 years. Those with diabetes and no comorbid diagnosis comprised 10.6% of the sample. Hypertension was the most common medical comorbidity (78.2%) and depression was the most common psychiatric comorbidity (12.7%). Over one fifth of the population (22.5%) had three or more medical comorbidities and 3.2% of the population had two or more psychiatric comorbidities. During the follow-up period, 21.9% of individuals in the cohort died. Table 1 shows demographic and comorbidity profile of the study population. The average survival time of those that died was 1.97 years. About one quarter of deaths occurred in each of the first (25.8%) and second (26.2%) year of follow-up with 9.1% occurring in the final year of follow-up.

**Table 1:** Demographic and Comorbidity Profile of Veterans*

**Demographic Variables**	**All (n = 625,903)**	**Alive (n = 488,287)**	**Deceased (n = 137,616)**
Age (years; mean, sd)	65 (11.1)	64 (10.9)	71 (9.8)
Male	97.8	97.5	98.7
Race/Ethnicity			
Non-Hispanic Black	72.1	69.9	79.8
Non-Hispanic White	13.2	13.5	12.3
Hispanic	5.3	5.5	4.6
Other/Unknown race	9.4	11.1	3.3
Married	65.3	66.1	62.3
Area of Residence			
Rural	36.8	36.7	37
Urban	61.7	61.8	61.6
Highly rural	1.5	1.5	1.4
Service Connection <50%	87.7	87.8	87.6
Geographic Region			
Northeast	11.0	11.0	11.1
Mid-Atlantic	21.7	21.6	21.9
South	21.1	21.0	21.5
Mid-West	30.9	31.0	30.7
West	15.3	15.4	14.8
Medical Comorbidity			
Hypertension	78.2	77.4	81.2
Lung disease	13.8	11.2	23.0
Obesity	12.9	13.6	10.2
Peripheral vascular disease	11.9	9.4	20.8
Cerebrovascular disease	11.4	9.1	19.7
Congestive heart failure	11.2	7.4	24.5
Anemia	7.5	5.6	14.0
Cancer	7.3	5.6	12.6
Hypothyroidism	6.2	5.8	7.5
Fluid/electrolyte disorders	4.9	3.6	9.6
Other disease	3.7	2.8	6.6
Cardiovascular disease	3.6	2.7	6.8
Liver disease	3.1	2.5	5.5
Psychiatric Comorbidities			
Depression	12.7	12.5	13.6
Psychoses	4.4	4.2	5.1
Substance abuse	3.9	3.7	4.5
Medical Comorbidities			
0 Medical comorbidity	12.1	13.8	6.4
1 Medical comorbidity	38.1	41.8	24.8
2 Medical comorbidities	27.3	27.1	27.9
3+ Medical comorbidities	22.5	17.3	40.9
Psychiatric Comorbidities			
0 Psychiatric comorbidity	82.8	83.4	80.8
1 Psychiatric comorbidity	14.0	13.5	15.8
2+ Psychiatric comorbidities	3.2	3.1	3.4
Total Comorbidities			
1 Comorbidity	34.0	37.3	22.0
2 Comorbidities	27.2	27.5	26.1
3 Comorbidities	14.9	13.6	19.7
4 Comorbidities	7.4	5.9	12.6
5+ Comorbidities	6.0	3.8	13.9

*All numbers represent percentages unless otherwise indicated.

**Figure 1:**
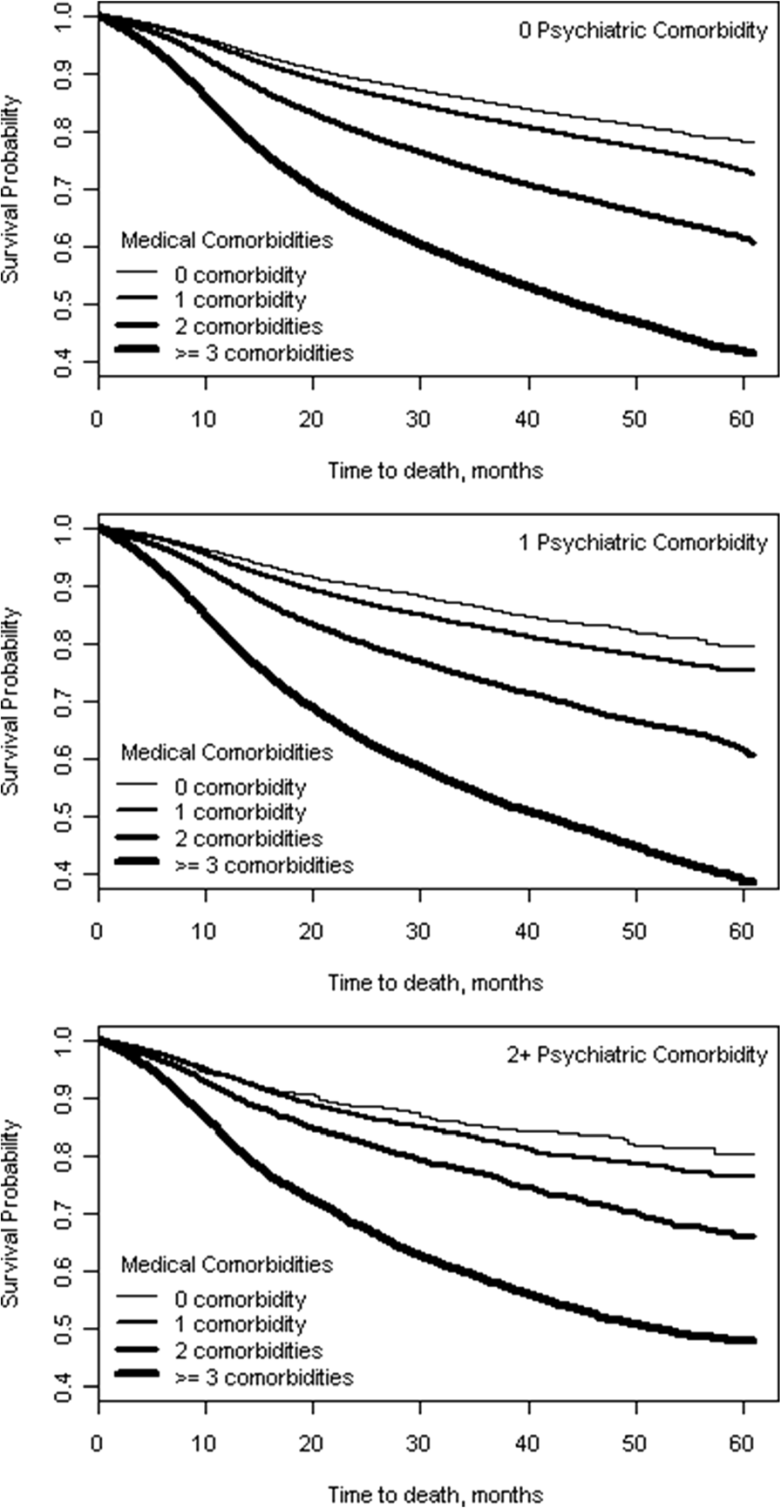
**Unadjusted Kaplan**-**Meier Survival Curves for Medical Comorbidity at Each Level of Psychiatric Comorbidity.**

**Figure 2:**
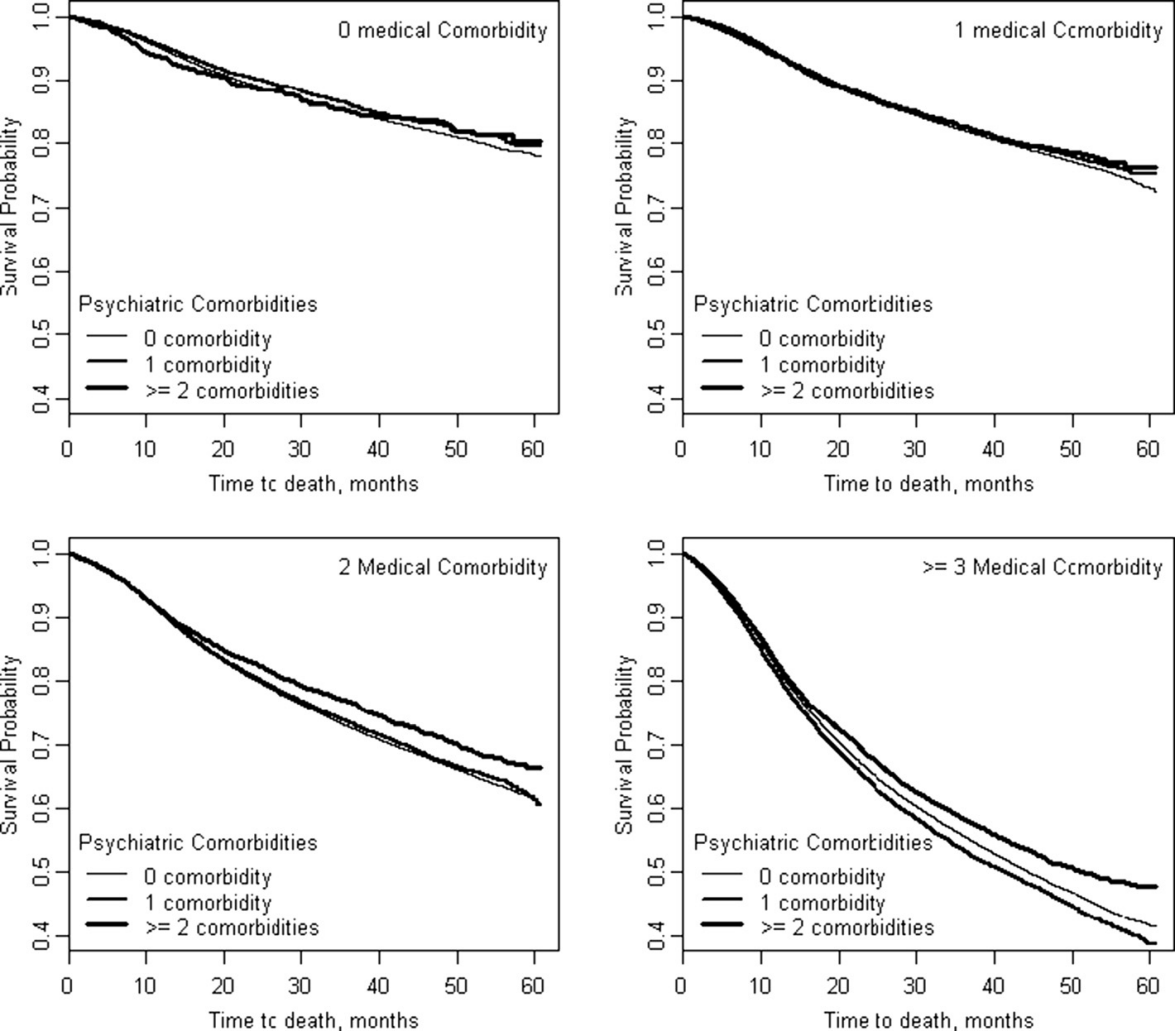
**Unadjusted Kaplan**-**Meier Survival Curves for Psychiatric Comorbidity at Each Level of Medical Comorbidity**.

The mortality risk associated with individual medical and psychiatric comorbidities after adjusting for demographic factors (age, gender, race/ethnicity, marital status, area of residence, service connection, and geographic region) are presented in Table 2. Of the medical comorbidities examined, congestive heart failure, which was present in 11.2% of the population, carried the greatest mortality risk with patients with congestive heart failure being almost twice as likely to die as those without congestive heart failure (HR = 1.92). Lung and cerebrovascular disease present in 13.8% and 11.4%, respectively, of the population also carried high mortality risk with patients with lung disease having 42% greater risk of mortality than those without lung disease and patients with cerebrovascular disease having 39% greater risk of mortality than those without cerebrovascular disease. Alternatively, hypertension and obesity each carried a lower mortality risk. For each of the psychiatric comorbidities examined, depression, psychoses and substance abuse carried significant mortality risk of 5%, 16% and 50%, respectively.Unadjusted Kaplan-Meier survival curves presented in Figure 1, illustrate the effect of medical comorbidity burden on probability of survival stratified by number of psychiatric comorbidities (i.e., 0, 1 and 2). Within each strata of psychiatric comorbidity burden, there was a clear graded relationship between number of medical comorbidities and probability of survival. Specifically, within each strata probability of survival was highest in those with zero medical comorbidities and lowest in those with 3 or more medical comorbidities. In Figure 2, unadjusted Kaplan-Meier survival curves illustrate the effect of psychiatric comorbidity burden on probability of survival stratified by number of medical comorbidities (i.e., 0, 1, 2 and 3 or more). In contrast to different survival curves for each level of comorbidity, Figure 2 illustrates a similar probability of survival across levels of psychiatric comorbidity burden in veterans with zero and 1 medical comorbidity. Moreover, while there is some differentiation in survival across levels of psychiatric comorbidity burden in veterans with 2 and 3 or more medical comorbidities, survival probability did not clearly decrease with increased psychiatric comorbidity burden.

**Table 2:** **Hazard Ratio and 95**% Confidence Interval Estimates for Each Disease Category* (**ordered by decreasing level of risk**)

	**HR (95% CI)**
Medical Comorbidities	
Congestive heart failure	1.92 (1.89, 1.95)
Liver disease	1.61 (1.57, 1.65)
Lung disease	1.42 (1.40, 1.44)
Cerebrovascular disease	1.39 (1.37, 1.40)
Cancer	1.38 (1.36, 1.40)
Other diseases	1.38 (1.35, 1.41)
Peripheral vascular disease	1.35 (1.33, 1.37)
Fluid/electrolyte disorders	1.33 (1.30, 1.36)
Anemia	1.25 (1.23, 1.27)
Cardiovascular disease	1.16 (1.13, 1.18)
Hypothyroidism	0.99 (0.97, 1.01)
Hypertension	0.91 (0.90, 0.92)
Obesity	0.88 (0.86, 0.90)
Psychiatric Comorbidities	
Substance abuse	1.50 (1.46, 1.54)
Psychoses	1.16 (1.14, 1.19)
Depression	1.05 (1.03, 1.07)

*Adjusted for sociodemographic characteristics including age, gender, race/ethnicity, marital status, area of residence, service connection, and geographic region.

Table 3 shows the HR and corresponding 95% CI for the association between number of medical comorbidities (i.e., 0, 1, 2 and 3 or more) and mortality stratified by number of psychiatric comorbidities (i.e., 0, 1 and 2) after adjusting for demographic factors (age, gender, race/ethnicity, marital status, area of residence, service connection, and geographic region). The mortality hazards associated with having 2 or 3 or more medical comorbidities as compared to no medical comorbidities were 1.54 and 2.63, respectively, in veterans with diabetes without any psychiatric comorbidity. Similar patterns in mortality hazards were seen in veterans with a single psychiatric comorbidity among those with 2 or 3 or more medical comorbidities compared to those with no medical comorbidities (HRs of 1.51 and 2.66, respectively). The difference in mortality hazard among those with greater medical comorbidity decreased only slightly in veterans with two psychiatric comorbidities (HR 1.30) compared to those with no medical comorbidity (HR 2.15), respectively.

**Table 3:** **Hazard Ratios** (**HR**)* **and 95**% **confidence Interval** (**CI**) **for Effect of Medical and Psychiatric Comorbidity Burden on Mortality among Veterans with Diabetes**

	**HR (95% CI)**
0 Psychiatric Comorbidities^a^	
0 Medical Comorbidity	1.00
1 Medical Comorbidity	1.05 (1.03, 1.08)
2 Medical Comorbidities	1.54 (1.51, 1.58)
3+ Medical Comorbidities	2.63 (2.57, 2.70)
1 Psychiatric Comorbidity^a^	
0 Medical Comorbidity	1.00
1 Medical Comorbidity	1.06 (0.98, 1.14)
2 Medical Comorbidities	1.51 (1.41, 1.63)
3+ Medical Comorbidities	2.66 (2.49, 2.85)
2 Psychiatric Comorbidities^a^	
0 Medical Comorbidity	1.00
1 Medical Comorbidity	1.02 (0.87, 1.19)
2 Medical Comorbidities	1.30 (1.12, 1.52)
3+ Medical Comorbidities	2.15 (1.86, 2.48)
0 Medical Comorbidities^b^	
0 Psychiatric Comorbidity	1.00
1 Psychiatric Comorbidity	1.23 (1.14, 1.32)
2+ Psychiatric Comorbidities	1.69 (1.47, 1.94)
1 Medical Comorbidity^b^	
0 Psychiatric Comorbidity	1.00
1 Psychiatric Comorbidity	1.23 (1.19, 1.27)
2+ Psychiatric Comorbidities	1.63 (1.52, 1.75)
2 Medical Comorbidities^b^	
0 Psychiatric Comorbidity	1.00
1 Psychiatric Comorbidity	1.20 (1.17, 1.24)
2+ Psychiatric Comorbidities	1.42 (1.34, 1.51)
3 Medical Comorbidities^b^	
0 Psychiatric Comorbidity	1.00
1 Psychiatric Comorbidity	1.24 (1.22, 1.27)
2+ Psychiatric Comorbidities	1.38 (1.32, 1.43)

*Interaction terms were used to determine adjusted hazard ratios for the effect of medical comorbidity within strata of psychiatric comorbidity^a^ as well as for the effect of psychiatric comorbidity within strata of medical comorbidity^b^. Adjusted for sociodemographic characteristics including age, gender, race/ethnicity, marital status, area of residence, service connection, and geographic region.

Additionally, Table 3 shows the HR and corresponding 95% confidence intervals (CI) for the association between number of psychiatric comorbidities (i.e., 0, 1 and 2) and mortality stratified by number of medical comorbidities (i.e., 0, 1, 2 and 3 or more) after adjusting for demographic factors. The mortality hazard associated with having a single as compared to zero psychiatric comorbidities remained constant across all medical comorbidity categories with respective hazard ratios of 1.23 and 1.24 in veterans with zero and 3 or more medical comorbidities. In contrast, the mortality hazard associated with having two as compared to zero psychiatric comorbidities decreased from 1.69 in veterans with zero medical comorbidities to 1.38 in veterans with 3 or more medical comorbidities.

## Conclusions

The findings of this study clearly demonstrate that the total burden of medical and psychiatric multi-morbidity are significant predictors of mortality among middle age and older adults (veterans) with type 2 diabetes with a graded response as medical multimorbidity increases. The average survival time was about 2 years with 52% of deaths occurring in the first 2 years of follow-up and 9% in the final year of follow-up. Among medical comorbidities, mortality risk was highest in those with congestive heart failure, lung disease and cerebrovascular disease; while among psychiatric comorbidities, mortality risk was highest in those with substance abuse, psychoses and depression. There was an interaction between medical and psychiatric comorbidity indicating that the association between mortality and medical comorbidity was modified by the presence of psychiatric comorbidity so stratified analyses were performed. HRs for effect of 3+ medical comorbidities remained high across levels of psychiatric comorbidities (0, 1, 2+), while HRs for effect of 2+ psychiatric comorbidities declined across levels of medical comorbidity (0, 1, 2, 3+).

Interestingly, comorbid hypertension and obesity were associated with lower mortality risk in this sample of veterans with type 2 diabetes. Since more than two-thirds of obese veterans also have comorbid hypertension [17], it is likely that the reduced mortality risk associated with hypertension was demonstrated among obese veterans. These findings may be explained by multiple interventions within the VA system from 2000–2010 (encompasses the time period of this study) that were successful in achieving blood pressure control among veterans who are provided access to healthcare services and to a very affordable formulary of anti-hypertensive medications [18]. Furthermore, one should consider that this analysis examined data generated shortly after diabetes was designated as a CVD risk equivalent along with tobacco smoking, hypertension, and hyperlipidemia [19]. In that case, it is likely that a strong awareness of CVD as a leading cause of death, growing evidence of behavioral management and treatment effectiveness from multiple large-scale RCTs (like Hypertension Optimal Treatment (HOT), United Kingdom Prospective Diabetes Study (UKPDS), Diabetes Prevention Program (DPP), Action to Control Cardiovascular Risk in Diabetes (ACCORD), and Antihypertensive and Lipid Lowering Treatment to Prevent Heart Attack Trial (ALLHAT), to name a few), and broad public health efforts lead by guidelines from several national organizations (American Heart Association (AHA), American Diabetes Association (ADA), and National Heart, Lung, and Blood Institute (NHLBI)) to aggressively manage CVD risk factors among people with diabetes not only reduced CVD mortality rates, but also helped to reduce the impact of comorbid hypertension and obesity on mortality in people with diabetes.

Medical comorbidities exerted the greatest influence on mortality and had significantly stronger effects on hazard of death compared to psychiatric comorbidities. In stratified analyses, the risk of death with increasing medical comorbidity remained high across each level of psychiatric comorbidity with the greatest risk seen in individuals with 3+ medical comorbidities (HR ranged 2.15-2.66) compared to no psychiatric comorbidity. In contrast, risk of death with increasing psychiatric comorbidity was not consistently high across levels of medical comorbidities. Instead the risk of death with 2+ psychiatric comorbidities declined as the number of medical comorbidities increased. In other words, among those with 2 or more psychiatric comorbidities, baseline risk (no medical comorbidity) is higher while the risk associated with additional medical comorbidity is lower. One explanation could be increased care associated with multiple medical and psychiatric comorbidities. However, the mortality risk from psychiatric comorbidities remained significant, emphasizing the need for greater attention to identifying and treating psychiatric comorbidities, instead of focusing on medical comorbidities alone.

There is growing awareness of the burden of multi-morbidity and the need to develop new treatment paradigms that account for multi-morbidity. This is particularly important for certain populations such as the elderly, veterans and low income and ethnic minorities with diabetes, where the prevalence of multi-morbidity is high and the risk for adverse outcomes increase exponentially with increasing levels of multi-morbidity [20-22]. The current approach to treatment for diabetes that is focused on single or related disease (e.g. diabetes and CVD) management may no longer be appropriate based on emerging evidence of the high prevalence of multi-morbidity and its detrimental effect on health outcomes including polypharmacy, increased morbidity, disability, health utilization/cost and mortality [5,7,23-26]. As the paradigm of care shifts to patient-centered care, strategies are needed to treat the individual as a whole and coordinate care while accounting for medical and psychiatric multi-morbidity. This will be particularly important in middle age and older adults, where our findings show that multi-morbidity is associated with significant increases in mortality. Newer evidence suggests that interventions linked to care delivery and focused on specific patient needs (e.g., medication management or functional status) are effective for improving outcomes among patients with multi-morbidity [7]. Our findings further highlight the need to integrate care for medical and psychiatric conditions and address the fragmentation of health care that currently exists with separate coverages for medical and mental health conditions. Some of the strategies will require policy change in how mental health services are covered and reimbursement structure in primary care settings.

Strengths of our study include the study population, its longitudinal design with 5 years of follow-up data, the extensive data available on comorbidities, and our ability to identify racial/ethnic group in over 90% of the cohort. To place this study in context, we report on the limitations. First, the VA medical record either omits or fails to update many important social, environmental and behavioral variables including socio-economic status, diet, physical activity, weight and alcohol consumption, as well as disease measures such as diabetes duration. Second, there is no way to account for the lag time between disease onset and diagnosis; thus, the timing of onset of comorbidity cannot be accurately determined in this retrospective cohort analysis. Finally, there were no measures of disease control accounted for so the findings are generalizable to those veterans with the diagnoses included in this study.

Nevertheless, our findings are important and show that, in this national longitudinal cohort of veterans with diabetes followed over 5 years, multi-morbidity was associated with increased risk of death and the risk of death was incremental with a graded response in mortality risk with increasing numbers of medical and psychiatric comorbidities.

## Competing interests

The authors declare that they have no competing interest.

## Authors’ contributions

LE conceived study concept and design. MG and LE were responsible for acquisition of data. CL, LE, MG, YZ, and KH analyzed and interpreted data. CL, KH, and MG drafted the manuscript. Critical revision of the manuscript for important intellectual content was provided by CL, KH, and LE. Study supervision was provided by LE. All authors read and approved the final manuscript.

## Pre-publication history

The pre-publication history for this paper can be accessed here:

http://www.biomedcentral.com/1472-6823/14/68/prepub
